# Resurrection and resilience of the rarest butterflies

**DOI:** 10.1371/journal.pbio.2003488

**Published:** 2018-02-06

**Authors:** Nick M. Haddad

**Affiliations:** Kellogg Biological Station, Department of Integrative Biology, Hickory Corners, Michigan, United States of America

This Perspective is part of the C*onservation Stories from the Front Lines Collection*

For as long as I can remember, I have been attracted to things that are rare. As a child, these things included coins, stamps, and football cards. When I was invited as a young professor to lead efforts in science and conservation of one of the world’s rarest butterflies, I accepted enthusiastically. Beginning in 2002, I focused my efforts on conservation and recovery of St. Francis’ Satyr (*Neonympha mitchellii francisci*) and its small remaining populations. I am by nature an over-optimist, and I knew (or hoped anyway) I could succeed and conserve the butterfly quickly.

The only place in the world that St. Francis’ Satyr can be found is in southern North Carolina. It has required active conservation since the subspecies’ discovery in 1983 at the Fort Bragg Army Installation. At that time, its total worldwide population was thought to number 100 butterflies in one population that occupied 1 ha in area [1]. By 1990, the population had declined to zero, and the subspecies was declared extinct (left half of Fig 1A) [2].

**Fig 1:**
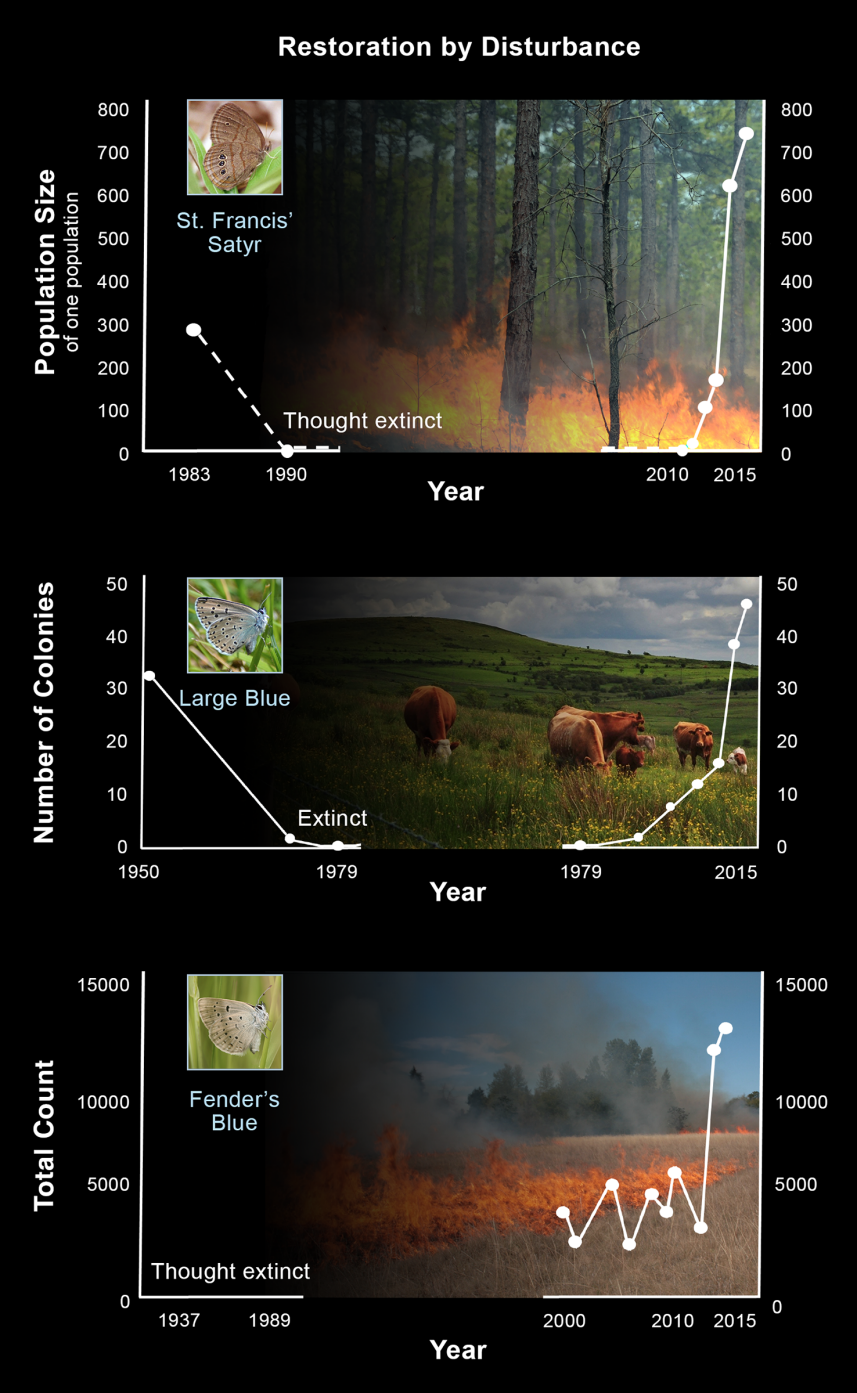
Population sizes for (a) St. Francis’ Satyr (S1 Data), (b) Large Blue in England data taken from [7], and (c) Fender’s Blue data taken from [8]. (Image Credit [top to bottom]: Brian Hudgens, Elizabeth J Evans/Fort Bragg, Peter Law, Bobby McKay, Donald Gudehus, Public Domain).

St. Francis’ Satyr’s resurrection was nearly as surprising as its original discovery. In 1993, Fort Bragg biologist Erich Hoffmann discovered a population in the most inaccessible place, the range where the army fires guns, flares, and heavy artillery. With this new knowledge and with a wider search of the installation, Hoffmann found St. Francis’ Satyr to occupy 10 ha of wetlands. Although a tiny area, it provided an apparently stable starting point from which my lab could work to expand and grow populations.

St. Francis Satyr’s physical features are unremarkable. It is small and brown. Most frequently, I see individuals sitting low on blades of grass. The butterfly lives along streams in open, grassy wetlands. A common assumption in conservation is that if we can exclude people from wild places, those landscapes—and the populations they support—will recover. So I set out to keep people, including scientists and soldiers, out of the way. I set up initial hands-off prescriptions, sat back…and watched the populations slide toward extinction.

I am at the same time bemused and appalled as I look back on my initial self-assuredness. Of seven populations that my group monitored outside artillery ranges, I watched as one was extirpated in 2003, the next in 2005, and the next three in 2008, 2009, and 2010 [3]. When I began my efforts in 2002, the population size of St. Francis’ Satyr reached 1,500. By 2011, it had descended to 100 [4]. Despite my effort and oversight, extinction appeared inevitable.

As I watched populations collapse, there was one thing that nagged at me. St. Francis’ Satyr’s saving grace is that most of its total range is confined within areas that at first seem hostile: artillery ranges. Because these areas are nearly completely closed to access, the reasons they harbored St. Francis’ Satyr had remained a mystery. Knowing butterflies were in there, my lab could create hypotheses that might explain their presence, hypotheses that were all poorly supported. How could St. Francis’ Satyr possibly thrive amid the cacophony of artillery?

My lucky break came just as St. Francis’ Satyrs were in their sharpest decline. It was then that I was granted access to the artillery ranges for the very first time. When I first entered, I nervously made my way for kilometers through the shards of casings scattered about the landscape. Yet it wasn’t the danger of the debris that was at the top of my mind. With the impending collapse of St. Francis’ Satyr populations beyond the artillery ranges, I was thrilled by my first encounters with the burgeoning populations.

On my first trip in, I was awed by the ranges’ unique habitats. Unlike habitats outside, the ranges are wide open woodlands harboring rare plants and animals. Whereas wetlands outside the ranges are thick with trees, shrubs, and vines, the wetlands inside are maintained wide open, conditions ideal for St. Francis’ Satyr. Problems with maintaining these conditions outside the ranges are overcome within them by two forms of disturbance. Artillery replicates historical and natural fire regimes, igniting vegetation and spreading unimpeded by infrastructure. Beaver (because they are not considered pests) create long networks of flooded wetlands. Together, bombs and beavers create open wetlands and butterflies.

My visits to the artillery ranges coupled with near extinction outside finally broke me from my hands-off approach to restoration. I now recognized that more, not less, disturbance is needed. Wetland habitats change over time, as natural disturbance resets vegetation before succession transforms grassy wetlands to forest. For a butterfly whose caterpillars eat sedges, trees are a destructive force. Paradoxically, so are disturbances caused by beaver and fire [3, 4]. Butterflies cannot survive flooding, fires, or succession. For populations to survive, I reasoned, they must disperse along stream corridors to move from degrading to improving habitats [5]. Even though the disturbance may harm one isolated butterfly population, it quickly generates high-quality habitats that increase those populations in the longer term. This variety of disturbed and recovering habitats across the landscape increases the entire population across St. Francis’ Satyr’s range. This caused me to propose disturbances that replicate range-like conditions in new areas.

Without artillery at our disposal, my lab became ersatz beavers and began recovery of St. Francis’ Satyr habitat. Working where there were no St. Francis’ Satyrs, we created an experiment to test effects of different types of disturbance caused by beaver [6]. In some plots, we removed trees. This could be done by brute force, as we felled trees with chainsaws and hauled them out by hand. Dam creation is more challenging, both in materials and sizes of dams. We discovered a company that could make custom-size dams that we could install easily by inflating them with water from a river or stream. Our dams were 150 feet long by 18 inches high, a size meant to impede water flow and create grassy wetlands hospitable for the butterfly.

We installed our experiment in 2011. One block was located 200 m from an existing St. Francis’ Satyr population, and the experimental sites were colonized immediately. The other block was distant from existing populations, and we had to seed it with butterflies raised in a research greenhouse.

Populations began to grow. In successive years, population sizes rose to 50 then 100 then 200 butterflies (Fig 1a). Today, the population size in our experimental restoration sites has reached 750 butterflies. When analyzed together with the artillery ranges, our restored area now supports one-fifth of the global St. Francis’ Satyr population. Success here has given me hope that we can restore other places on and off the military installation. My 15-year effort now seems to be bearing fruit in the sense that we are observing the first signs of recovery.

And then I learned a harder lesson: while playing a pickup basketball game three years ago, I fell, cracked my head, and sustained a severe traumatic brain injury. I lost a period of my life. My first recollections were two months later, looking up from a bed in an acute rehabilitation hospital. It was easy for me to stumble around the unit, see and interact with other patients, and believe I was now “fine.” For two months without memory and many more months of recovery, my family and close friends helped me in some ways that I know and others that I will never know. St. Francis’ Satyr also helped in my recovery, as the butterfly gave and guided my focus toward the power of restoration, both of my life and of nature.

A short time after I’d regained my memory, I encountered one of the many nurses who cared for me in the intensive care unit. The first thing Zandro asked was this: “Nick, how are those rare butterflies?” I wondered out loud: how did you know about my interest in rare butterflies? He responded, “You talked about them every 90 seconds for three straight days.” That comment was at the same time amusing and profound. In an unexpected way, it reinforced how central those rare butterflies had become to my work and my life.

Through months of needed recovery, I had idle time to reflect on many things, including St. Francis’ Satyr. I was eventually able to look with fresh eyes at data we had collected in the years before and after our restoration efforts (Fig 1). I looked at graphs of butterfly numbers over time, the same graphs I’d looked at tons of times before, and saw two different patterns. Before restoration, population size fell rapidly toward zero (left half of Fig 1a). The magnitude and rate of decline were frightening until the population was extirpated. When I had proposed restoration, I’d viewed it primarily as a test of principles of basic ecological science. By the time restoration had begun, the population had descended to such a low level that the need for restoration success had grown urgent.

Then, in the same graphic, I observed resurrection (right half of Fig 1a). Restoration success was in no way certain. I watched the surprising speed at which the population recovered. Our efforts resulted in a rare success at the intersection of science and endangered species conservation [9].

At the onset of my recovery, my father-in-law reminded me of my book project. Days before my accident, I had submitted a proposal (likely the reason the nurse heard so much about rare butterflies in my early delirium). My father-in-law suggested that some of the best therapy during my recovery would be to begin writing that book. He was right. It took my own path to recovery to reveal to me the decline and resurrection of St. Francis’ Satyr and of other rare butterflies.

As I assembled case after case, species after species, I discovered three things. First, most of the rarest butterflies are becoming rarer (e.g., Fig 1 [10, 11, 12]). Second, nearly all of the rarest butterflies are dependent on natural disturbance. I assembled classic case studies (Fig 1). In one, after over a century of scientists anguishing about its conservation, the Large Blue in southwest England (*Phengaris arion eutyphron*) went extinct. Simultaneously, scientists learned that herbivory was needed to create grasslands suitable to the butterfly’s mutualist ant [7, 13, 14]. Only after overcoming fear of the harm cows might cause butterfly populations could new restoration include grazers. With this knowledge, the reintroduction in England of another Large Blue subspecies from Sweden has been a remarkable success (Fig 1c). In another case, Fender’s Blue (*Icaricia icarioides fenderi*) in the Willamette Valley in Oregon was thought to be extinct for fifty years. Its rediscovered populations were small and declining. It was impossible to imagine restoring fire to incinerate plants and butterflies. As scientists and conservationists watched, small prairies succeeded to shrubland. Only after the introduction of regular fire to targeted parts of the butterfly’s range each year did habitats improve in quality and the butterfly population grow (Fig 1b [8, 15]).

I want to be clear and distinguish natural from human-caused disturbance. In a meta-analysis of long-term Lepidopteran studies globally, Dirzo et al. [16] found consistent, strong decreases in abundance and diversity in response to disturbances caused by agriculture, urbanization, forestry, and others. Natural disturbances, however, maintain the open habitats in which many rare butterflies live. These areas include grasslands kept open by fire [17] or by herbivory [13, 18, 19], wetlands kept open by flooding [4], coastal dune formation [20], and forest boundaries kept by storms [21]. Disturbance management must be a strong part of the conservation repertoire for rare butterflies.

My third discovery was that decline was reversed only after recognition of one uncomfortable and seemingly incompatible truth: we have to kill some butterflies to save butterflies (Fig 1). By allowing or even creating disturbance in some fraction of the habitats where there are rare butterflies, some are killed now [3, 4, 15, 22]. Simultaneously, disturbance that is at first harmful then rehabilitates habitats to support populations going forward. A caveat is that all populations cannot be disturbed simultaneously. Large and persistent metapopulations must encompass areas with butterflies that produce colonists for new habitats. Restricting disturbance to prevent harm is akin to *Of Mice and Men*’s Lennie squeezing a puppy [23]—loving populations to death.

Could lessons learned in conservation of the rarest butterflies be applied to other insects in decline? The Monarch population of eastern North America has plummeted by 80% or more in the last two decades [24]. A global analysis of insect abundances found that butterflies and moths have dropped 30% in the past four decades; all insects have declined by 70% [16]. Insect conservation requires halting or reversing habitat loss, the sure reason for most of these declines. The lesson learned from the rarest butterflies is that habitat loss includes the loss of natural ecosystem processes such as disturbance [25]. Recovery of the rarest butterflies, and assuredly for other insects, must include restoration of natural disturbance.

## Supporting information

S1 Data Annual estimates of population size of St. Francis’ Satyr in one restoration site. (XLSX)
